# Identification of Molecular Markers of Clozapine Action in Ketamine-Induced Cognitive Impairment: A GPCR Signaling PathwayFinder Study

**DOI:** 10.3390/ijms222212203

**Published:** 2021-11-11

**Authors:** Agata Korlatowicz, Maciej Kuśmider, Marta Szlachta, Paulina Pabian, Joanna Solich, Marta Dziedzicka-Wasylewska, Agata Faron-Górecka

**Affiliations:** Laboratory of Biochemical Pharmacology, Department of Pharmacology, Maj Institute of Pharmacology, Polish Academy of Sciences, Smętna Street 12, 31-343 Krakow, Poland; korlat@if-pan.krakow.pl (A.K.); splaknak@gmail.com (M.K.); marta.szlachta90@gmail.com (M.S.); palach@if-pan.krakow.pl (P.P.); solich@if-pan.krakow.pl (J.S.); wasyl@if-pan.krakow.pl (M.D.-W.)

**Keywords:** ketamine, clozapine, GPCRs, βarrestins, ERK1/2, Girk3, Grk2

## Abstract

Background: Cognitive disorders associated with schizophrenia are closely linked to prefrontal cortex (PFC) dysfunction. Administration of the non-competitive NMDA receptor antagonist ketamine (KET) induces cognitive impairment in animals, producing effects similar to those observed in schizophrenic patients. In a previous study, we showed that KET (20 mg/kg) induces cognitive deficits in mice and that administration of clozapine (CLZ) reverses this effect. To identify biochemical mechanisms related to CLZ actions in the context of KET-induced impairment, we performed a biochemical analysis using the same experimental paradigm—acute and sub-chronic administration of these drugs (0.3 and 1 mg/kg). Methods: Since the effect of CLZ mainly depends on G-protein-related receptors, we used the Signaling PathwayFinder Kit to identify 84 genes involved in GPCR-related signal transduction and then verified the genes that were statistically significantly different on a larger group of mice using RT-PCR and Western blot analyses after the administration of acute and sub-chronic drugs. Results: Of the 84 genes involved in GPCR-related signal transduction, the expression of six, *βarrestin1*, *βarrestin2*, galanin receptor 2 (*GalR2)*, dopamine receptor 2 (*DRD2)*, metabotropic glutamate receptor 1 (*mGluR1*), and metabotropic glutamate receptor 5 (*mGluR5*), was significantly altered. Since these genes affect the levels of other signaling proteins, e.g., extracellular signal-regulated kinase 1/2 (ERK1/2), G protein-coupled receptor kinase 2 (Grk2), and G protein-gated inwardly rectifying potassium 3 (Girk3), we determined their levels in PFC using Western blot. Most of the observed changes occurred after acute treatment with 0.3 mg/kg CLZ. We showed that acute treatment with CLZ at a lower dose significantly increased βarrestin1 and ERK1/2. KET treatment induced the upregulation of βarrestin1. Joint administration of these drugs had no effect on the βarrestin1 level. Conclusion: The screening kit we used to study the expression of GPCR-related signal transduction allowed us to select several important genes affected by CLZ. However, the obtained data do not explain the mechanism of action of CLZ that is responsible for reversing KET-induced cognitive impairment.

## 1. Introduction

One of the most serious neuropsychiatric illnesses is schizophrenia. Prefrontal cortex dysfunction is closely related to cognitive impairment [1]. These deficits appear early in the disease, persist without remission for most of the patients’ lives, and may precede the development of positive symptoms. The flexibility of attention shift in response to a set of stimuli is controlled by the PFC and is a parameter measured in humans by the Wisconsin Card Sorting Test [2]. This test is considered a tool to diagnose prefrontal cortex lesions. Moreover, most schizophrenic patients have problems with performing tasks that require the involvement of executive functions. This disturbance can also be tested in rodents using the attentional set-shifting task (ASST) [3,4].

In our previous study, mice were given ketamine (KET 20 mg/kg) to induce cognitive deficits and clozapine (CLZ) at two different doses (0.3 and 1 mg/kg) to reverse KET-induced cognitive deficits. The results of the experiment showed that the administration of CLZ reverses the cognitive deficits caused by KET, but this effect was only observed with the use of a lower dose of CLZ [5]. In a further study, we demonstrated the presence of heterodimers for dopamine DRD2 and serotonin 5HT1A receptors as well as serotonin 5HT1A and 5HT2A receptors in the prefrontal and frontal cortexes using the proximity ligation technique (PLA). Moreover, we showed a significant effect of CLZ (0.3 mg/kg) administration on the observed PLA signal. For the tested pair of 5HT1A–5HT2A serotonin receptors, we observed an increase in the interaction of these receptors after sub-chronic KET and CLZ (0.3 mg/kg) administration, and this effect was enhanced by concomitant administration of these drugs [6]. Despite the demonstration that CLZ acts through heterodimers of GPCRs [7,8,9], the biochemical data did not correlate with the observed behavioral effect.

Therefore, the main goal of the study was to identify the mechanism of action of CLZ in the context of KET-induced cognitive impairments. Demonstration of behavioral correlations with biochemical changes could suggest mechanisms associated with the unique effects of this drug.

CLZ is a dopamine (DRD2) and serotonin (5HT2A) antagonist with several unique differences from other atypical antipsychotic drugs. CLZ has much stronger antagonistic effects on cortical and limbic dopamine DRD4 receptors than DRD2 receptors. Moreover, it acts as an antagonist of serotonergic 5HT2 receptors (5HT2A and 5HT2C) and adrenergic (α1), histamine (H1), and muscarinic (M1) receptors [10]. It appears that the combination of relatively high DRD1, low DRD2, and very high 5-HT2 receptor occupancy is unique to CLZ and may explain its lower tendency to induce extrapyramidal side effects.

Since the effect of CLZ mainly depends on the presence of G protein-related receptors, we used the Signaling PathwayFinder Kit (Qiagen, Hilden, Germany) for screening, which allowed the identification of 84 genes (Table 1) related to GPCR involved in signal transduction and then verified statistically significant genes in a larger group of individuals using RT-PCR after acute and sub-chronic treatment.

Among the genes whose expression changed significantly during our study were the *βarrestins* (*1* and *2*). βarrestins are small cytosolic proteins that are traditionally known to be negative feedback regulators of GPCR [11]. However, more recent studies have shown that βarrestins can mediate intracellular signaling independent of their effect on G protein stimulation. By acting as scaffolding proteins, they can lead to the formation of intracellular signaling complexes that can regulate the functions of various signaling cascades, such as mitogen-activated protein kinase (MAP kinases), c-Jun N-terminal kinases (JNK), and nuclear factor kappa-light-chain-enhancer of activated B cells (NFκB), influencing gene expression [12,13].

These results point to new potential mechanisms of action not only for CLZ but also for KET. Although KET was used in our study as a tool to induce cognitive impairment, we also demonstrate important biochemical changes for this drug that may contribute to the elucidation of the mechanism of action of this drug.

## 2. Results

### 2.1. RT² Profiler™ PCR Array Mouse GPCR Signaling PathwayFinder

Since we have shown in our previous studies that acute CLZ at a dose of 0.3 mg/kg has a stronger effect than CLZ (1 mg/kg) for reversing KET-induced cognitive impairment in the ASST test in mice, we performed an initial screening test on this administration paradigm. The analysis was performed on mice that received a single dose of saline, KET (20 mg/kg), CLZ (0.3 mg/kg), or both KET (20 mg/kg) and CLZ (0.3 mg/kg).

**Figure 1:**
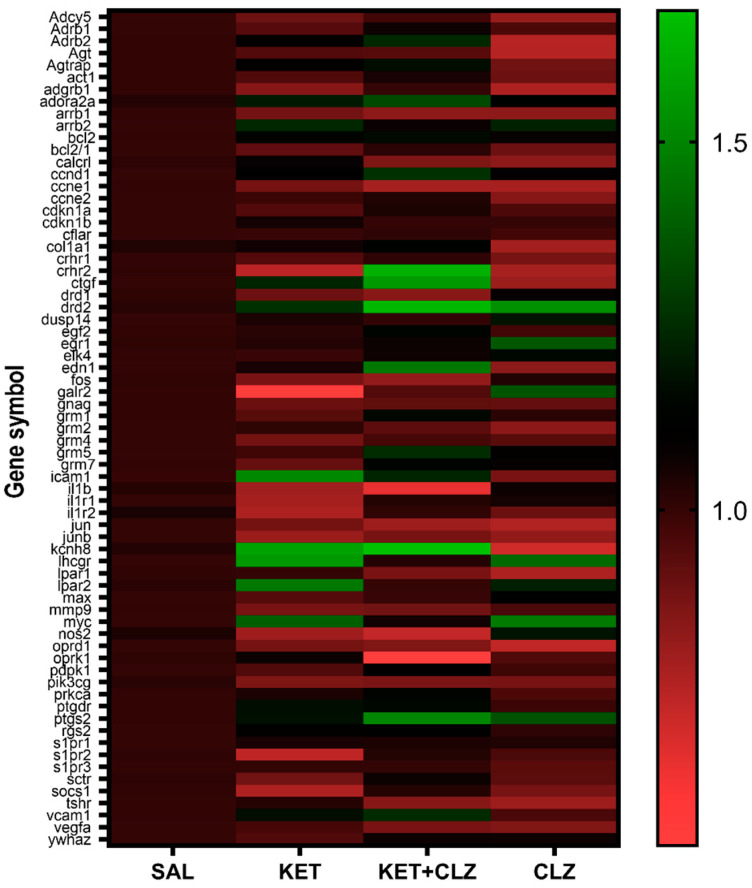
Relative mRNA Quantification (RQ) Heat Map after acute treatment with KET (20 mg/kg), CLZ (0.3 mg/kg), or both. The highest values are shown in green, and the lowest values are shown in red. Genes that changed statistically significantly were used for further verification using TaqMan Primers (Table 2).

Of the 89 genes identified (Table 1), 16 were not expressed (ct value > 35). Figure 1 presents the Relative mRNA Quantification (RQ) of the expressed genes. The statistical analysis using one-way ANOVA showed that nine genes were significantly regulated by the investigated drugs (*p* < 0.5). These genes were selected for further analysis (Table 2).

### 2.2. mRNA Expression Changes in the Prefrontal Cortex

**Figure 2:**
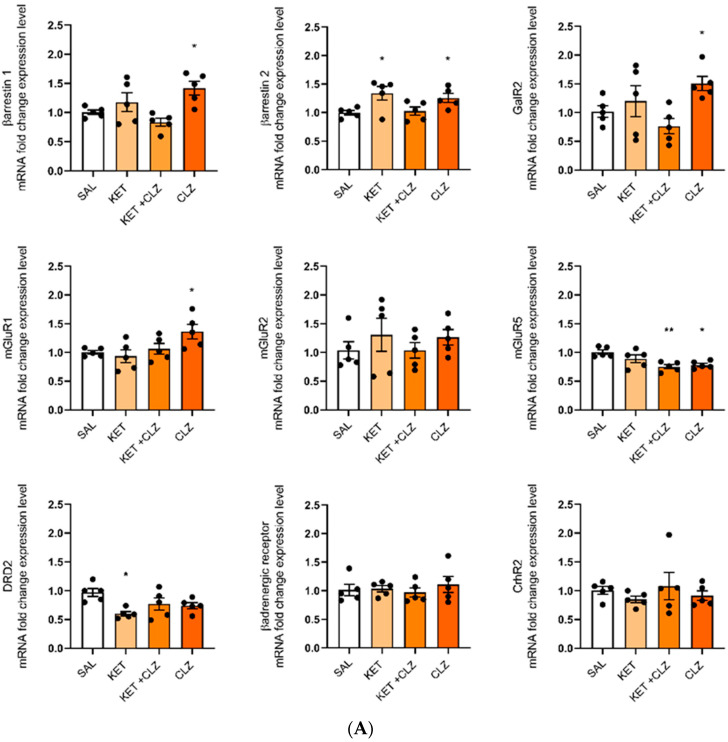

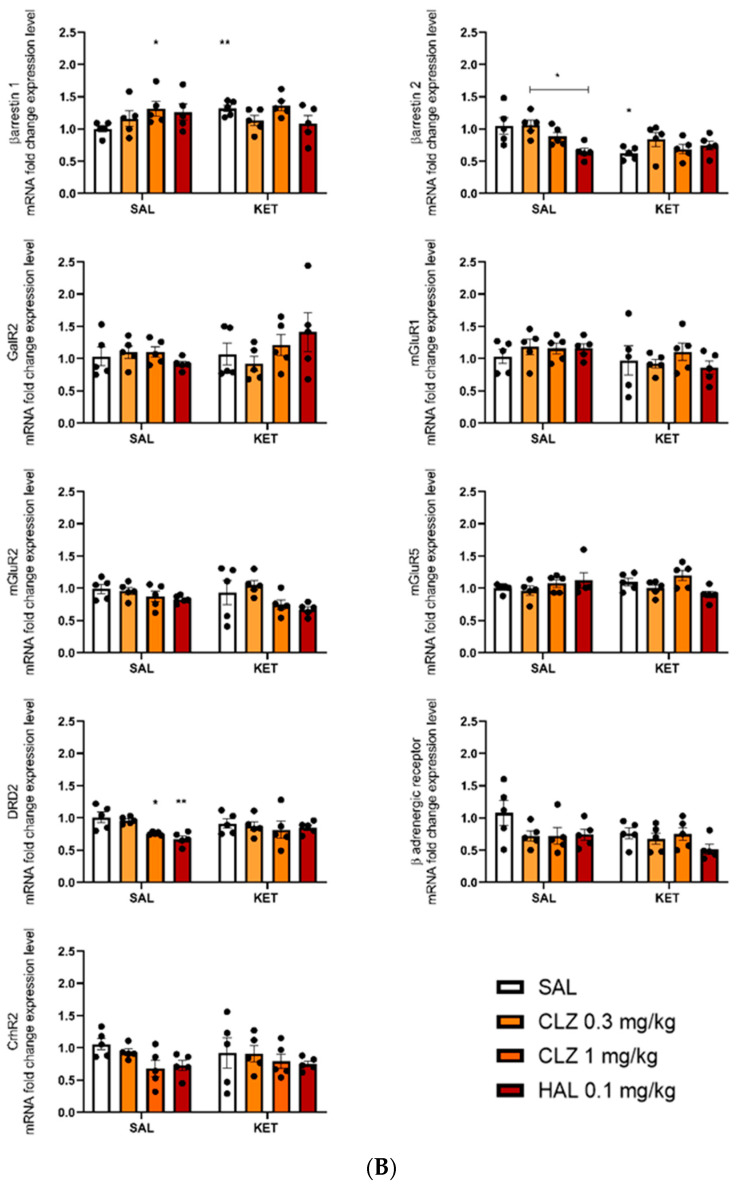
Expression of mRNA selected using the Signaling PathwayFinder Kit after the administration of KET, CLZ, HAL, or combinations of drugs. Results were obtained for n = 5 individuals with technical duplicates used for each group using TaqMan Primers (Table 2). The results are presented in the graph as mRNA expression fold changes. * *p* < 0.05, ** *p* < 0.01 vs. the control (SAL) group; (**A**) after acute treatment and (**B**) after sub-chronic treatment.

Since the RT2 qPCR analysis was conducted on only three mice, the results were verified using TaqMan probes (Table 2) on five individuals per group in technical duplicates. In the extended analysis, a significant difference in gene expression after acute treatment with CLZ (0.3 mg/kg) was observed for *mGluR1* (upregulation; *p* < 0.05) and *mGluR5* (downregulation; *p* < 0.05). KET treatment significantly downregulated the expression of *DRD2* (*p* < 0.05). Although the two-way ANOVA analysis showed no statistically significant change in *βarrestin2* expression after KET administration, Tukey’s post hoc analysis showed that KET significantly upregulated the expression level of this gene (*p* < 0.05). Moreover, the two-way ANOVA showed a statistically significant interaction between KET and CLZ. Co-administration of these drugs affected the expression levels of *βarrestin1* (F_(1,16)_ = 11.47; *p* < 0.01), *βarrestin2* (F_(1,16)_ = 12.07; *p* < 0.01), and *DRD2* (F_(1,16)_ = 7.742; *p* < 0.05). The observed interaction is not a result of synergistic actions of these drugs. The observed effects of CLZ and KET co-administration on the expression levels of these genes contrast with those observed for the single administration of these drugs (Figure 2A). Details of the statistical analysis can be found in Table 3.

**Figure 3:**
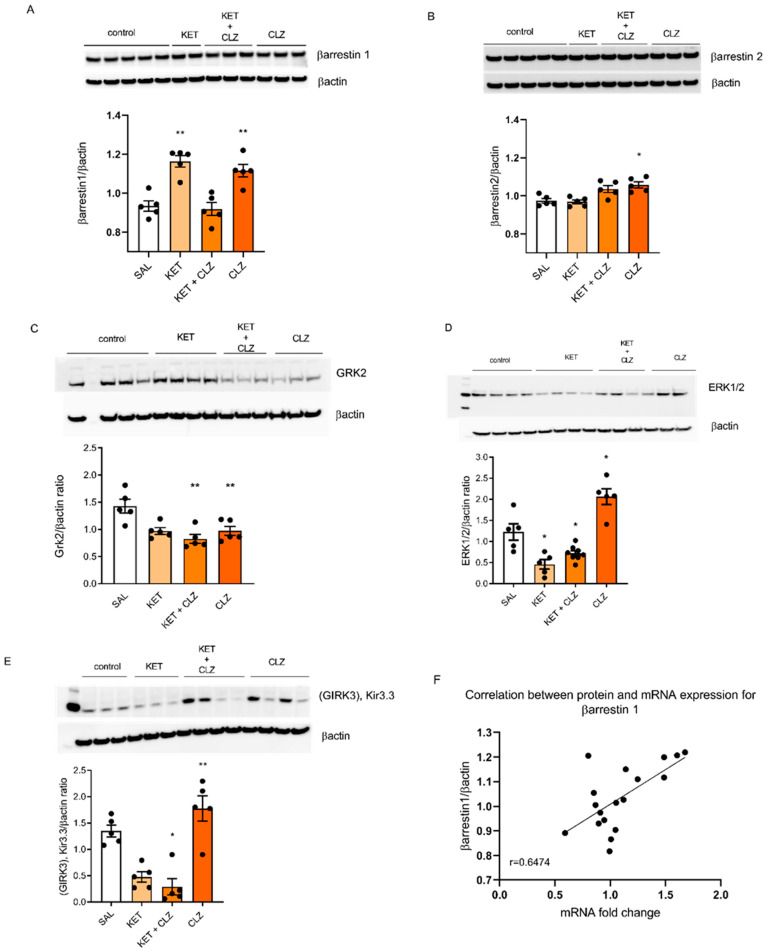
The effects of acute KET and CLZ administration on βarrestin1 (**A**), βarrestin2 (**B**), Grk2 (**C**), ERK1/2 (**D**), and Girk3 (**E**) levels in the PFC. (**F**) The graphs show the correlation between mRNA expression and the βarrestin1 protein level. Representative images of each protein and βactin are shown in the upper panels. Bars represent the mean ± S.E.M. * *p* < 0.5; ** *p* < 0.01 vs. the control group.

After sub-chronic treatment with CLZ, we did not observe statistically significant changes in the expression levels of the studied genes. However, Tukey’s post hoc analysis revealed significant upregulation of *βarrestin1* mRNA expression and downregulation of DRD2 (*p* < 0.05) with the higher dose of CLZ (1 mg/kg) (Figure 3B). As in our previous studies [6], we also included a typical neuroleptic, HAL (0.1 mg/kg), in the biochemical determinations. This was administered sub-chronically. A comparison of CLZ and HAL—drugs with different mechanisms of action—was conducted to provide additional information on the unique effects of CLZ. After sub-chronic administration of KET, we observed downregulation of *βarrestin2* (F_(1,24)_ = 14.41; *p* < 0.001). Moreover, a statistically significant interaction between KET and HAL (F_(1,16)_ = 10.800; *p* < 0.001) was demonstrated for the expression of this gene. A significant decrease in *DRD2* mRNA expression was observed after treatment with HAL (F_(1,16)_ = 9.375; *p* < 0.01). A two-way ANOVA showed a significant KET × HAL interaction for *DRD2* expression (F_(1,16)_ = 6.586; *p* < 0.05). Details of the statistical analyses can be found in Table 4 and Figure 2B.

### 2.3. Changes in the Levels of Selected Signaling Proteins

Because we observed statistically significant changes in the mRNA expression *of βarrestin1* and *βarrestin2* in both acutely and sub-chronically treated groups, we also measured the levels of proteins encoded by these genes. Moreover, due to the involvement of extracellular signal-regulated kinase 1/2 (ERK1/2), G protein-coupled receptor kinase 2 (Grk2), and G protein-gated inwardly rectifying potassium 3 (Girk3) proteins in the intracellular signal transduction of GPCRs, we also measured these proteins in all studied groups. Our results from the Western blot analysis demonstrated that a single administration of CLZ at a dose of 0.3 mg/kg induced most of the observed changes (Figure 3). The two-way ANOVA analysis showed a significant effect of the interaction between KET and CLZ on βarrestin1 after acute administration of these drugs (F_(1,16)_ = 41,21; *p* < 0.001). Independent treatment with these drugs caused an increase in the βarrestin1 protein level, while co-administration reduced this effect. The Tukey post hoc analysis showed significant increases in βarrestin1 (*p* < 0.01 (Figure 3A) and ERK1/2 (Figure 3D) levels (*p* < 0.05) after treatment with 0.3 mg/kg CLZ. The two-way ANOVA showed a significant impact of CLZ on βarrestin2 (F_(1,16)_ = 17.87; *p* < 0.001; Table 5; Figure 3B).

A single administration of KET caused a statistically significant decrease in the ERK1/2 (*p* < 0.05) and Girk3 (*p* < 0.05) protein levels but no change in Grk2. Although CLZ administered separately induced the upregulation of these proteins, co-administration of CLZ with KET resulted in a decrease in their levels, similar to the case of a single administration of KET. Thus, it seems that there is a site-of-action blocking effect by KET on CLZ. The statistical analysis showed that a single administration of CLZ significantly influenced the level of Grk2 (F_(1,16)_ = 23.35; *p* < 0.01; Figure 3).

**Figure 4:**
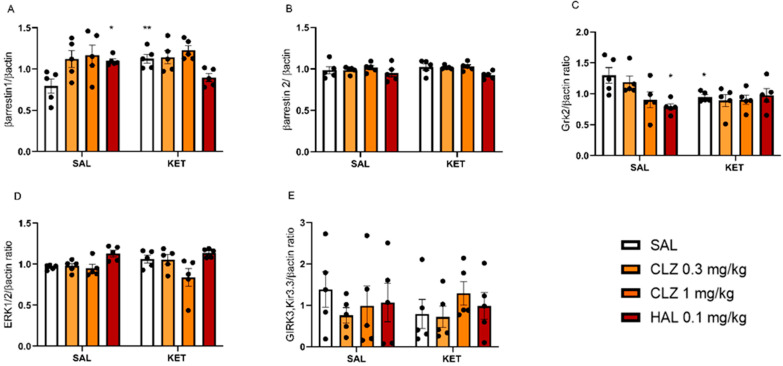

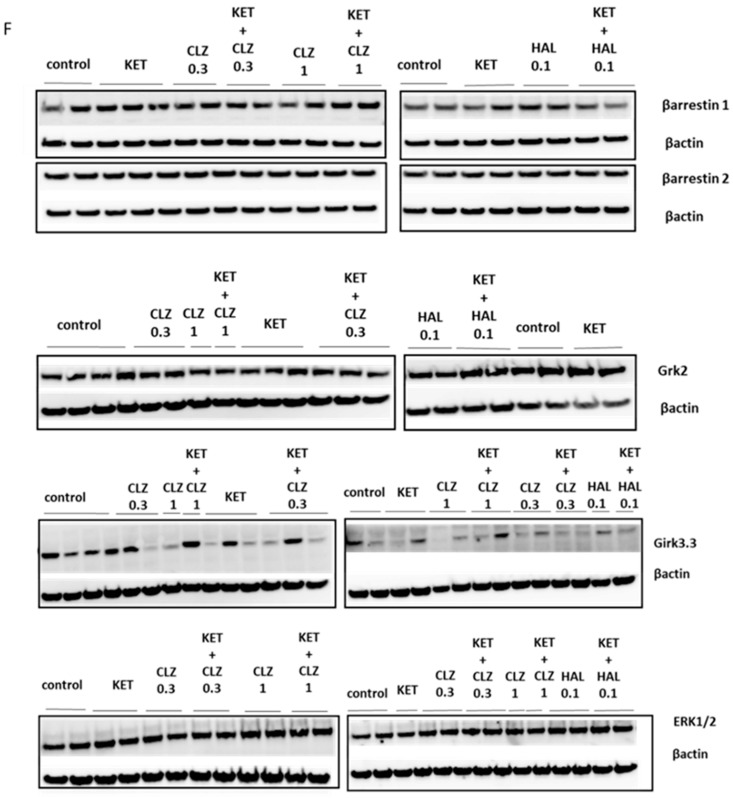
The effects of sub-chronic KET and/or CLZ or HAL administration on βarrestin1 (**A**), βarrestin2 (**B**), Grk2 (**C**), ERK1/2 (**D**), and Girk3 (**E**) levels in the PFC, as well as representative membranes of each protein and βactin (**F**). Bars represent the mean ± S.E.M. * *p* < 0.5; ** *p* < 0.01 vs. control group.

Surprisingly, the sub-chronic CLZ treatment did not induce significant changes in the levels of the studied proteins. Sub-chronic KET or HAL administration increased the concentration of the βarrestin1 protein (Figure 4A). An increase in the βarrestin1 level was also correlated with the observed increase in *βarrestin1* mRNA expression (r = 0.6474). In addition, a significant interaction of KET with HAL was observed F_(1,16)_ = 19.77; *p* < 0.001. In addition, a statistically significant decrease in the Grk2 level was observed after KET or HAL treatment. Details of the statistical analyses can be found in Table 6 and Figure 4.

## 3. Discussion

Many studies on animal models of schizophrenia have focused on the positive symptoms of the disorder, where the mesolimbic system plays a dominant role. In our earlier behavioral studies, we administered the non-competitive NMDA receptor antagonist KET, which causes cognitive impairment in animals, characteristic of that observed in patients with schizophrenia [14,15,16,17,18,19,20,21,22,23,24,25,26]. We showed that it induces cognitive deficits in mice and that administration of CLZ reverses this effect [5]. This result led us to search for biochemical correlates responsible for CLZ action.

In the present study, among the 84 studied genes involved in GPCR activity, the expression of only 9 changed significantly, and after verification with TaqMan arrays, only 6 (*βarrestin1*, *βarrestin2*, *DRD2*, *Gmr1*, *Gmr5*, and *GalR2*) were confirmed as showing changes. A second equally interesting finding from our analysis is that most of these changes occurred after acute administration of KET and/or CLZ drugs at a dose of 0.3 mg/kg, whereas chronic administration only resulted in changes in βarrestin1, βarrestin2, and DRD2.

### 3.1. Changes in Gene Expression after CLZ Administration

CLZ is an atypical neuroleptic, which means that dopamine DRD2 is not the most important element for its mechanism of action. However, studies have postulated that there is inverse agonism of atypical antipsychotics, including CLZ, on DRD2 [27,28]. Equally interesting, the loose-binding hypothesis postulates that CLZ acts by transiently blocking DRD2 [29]. Studies based on mathematical modeling of receptor occupancy also suggest that CLZ can block single DRD2-induced neuronal spikes, but it does not block spikes that mediate locomotion, cognition, and affect [30]. Moreover, in vitro studies on DRD2 overexpression indicate that CLZ binds to DRD2 with a high level of affinity [31]. Furthermore, the discovery of the action on GPCR receptor dimers has further broadened the search for CLZ’s unique action. In our previous studies, we showed that CLZ uncouples DRD1–DRD2 dopamine receptor dimers, and this effect is dose- and time-dependent. In addition, it also acts on serotonin 5HT1A, 5HT2A, and dopamine DRD2 receptor dimers. The affinity of CLZ to DRD1 and 5HT2A receptors depends on whether they are present in the plasma membrane separately or together with DRD2 [8,31]. In our previous study using the Proximity Ligation Assay (PLA), KET did not affect the 5HT1AR–DRD2 interaction, whereas when administered at low doses (0.3 mg/kg), CLZ increased the interaction of this receptor pair in the mouse brain [6]. These data suggest that DRD2 is also involved in CLZ’s mechanism of action and may be responsible for the unique effects of this drug. In the present study, we observed a decrease in DRD2 expression after chronic treatment with a higher dose of CLZ as well as after HAL administration (Figure 2B). Thus, it appears that CLZ, when given at a dose of 1 mg/kg, provides negative feedback to DRD2. This may support our earlier hypothesis that the effect of CLZ is strictly dose-dependent. The obtained results showing decreased DRD2 expression for CLZ and HAL are also reflected in the density of this receptor in the prefrontal cortex [6].

After acute treatment with CLZ at a dose of 0.3 mg/kg, an increase in galanin receptor 2 (GalR2) expression was observed, while no more changes were observed after chronic administration. It has been shown that GalR2 is involved in antidepressant effects. Mice overexpressing this receptor have been shown to exhibit antidepressant-like behaviors and increased resistance to stress [32]. On the other hand, there are also reports indicating that CLZ is effective for the acute and maintenance treatment of a bipolar disorder. Thus, it seems that GalR2 is responsible for one of the mechanisms associated with this action. Our results indicating the involvement of this receptor in the acute effects of CLZ are in line with these findings.

Administration of a low dose of CLZ also affects the expression of metabotropic glutamate receptors. Although the metabotropic receptors mGluR1 and mGluR5 belong to the same group of phospholipase C activating receptors, the administration of CLZ at a dose of 0.3 mg/kg had different effects on the expression of these receptors in the PFC. In our study, we observed an increase in mRNA mGluR1expression, while there was a decrease mGluR5 mRNA expression. There have been few reports on mGLuR1 and schizophrenia, but a role for mGluR5 in this disorder has been postulated. However, CLZ was shown to increase Ser831–mGluR1 phosphorylation in the PFC, and this was also the case for tianeptine [33]. Both drugs increase postsynaptic NMDA currents involving AMPA receptors [34]. Previous studies on the effects of antipsychotics on mGluR5 binding have shown inconsistent results. In rat studies, both HAL and sertindole were found to increase mGluR5 mRNA expression [35]. However, another study did not identify changes in mGluR5 protein expression following treatment with olanzapine and HAL [36]. In postmortem studies, no clear association between antipsychotic use and mGluR5 binding has been identified thus far [37]. Additionally, CLZ was analyzed in the PFC of Rhesus monkeys [38], and chronic treatment with CLZ was not found to affect mGluR5. No changes were observed in the cortexes of mice treated chronically with HAL [39]. These data are consistent with our results for the sub-chronic treatment of CLZ and HAL.

**Figure 5:**
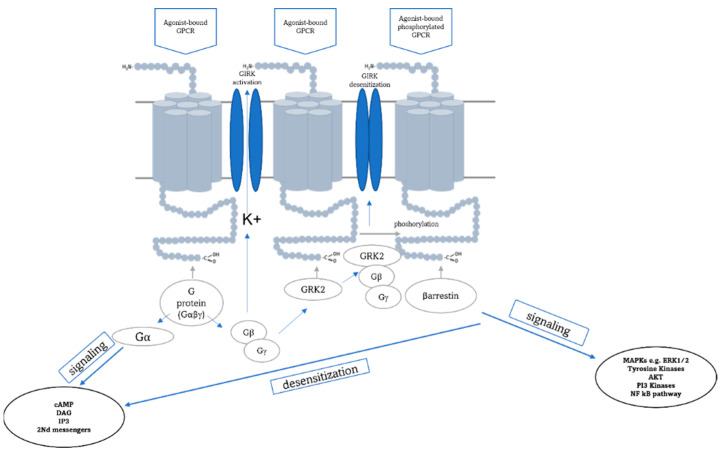
Schematic diagram of signal transduction by GPCRs. The active receptor stimulates heterotrimeric G proteins and is rapidly phosphorylated by G protein-coupled receptor kinases (GRKs), leading to the recruitment of βarrestins. βarrestins not only mediate the desensitization of G protein signaling but also act as signal transducers themselves, for example, by activating ERK1/2. Gβγ subunits bind and activate the GIRK channel. Due to the relatively higher affinity of Grk2 to Gβγ, Grk2 can effectively compete for the available pool of Gβγ with the GIRK channel, leading to the closure of the GIRK channel. (Based on [44,45]).

GPCRs undergo desensitization via activation-dependent phosphorylation by G protein-coupled receptor kinases (GRKs) followed by βarrestin binding. βarrestins and GRKs are major regulators of GPCR signaling. βarrestins and GRKs have been linked to mitogen-activated protein kinase (MAPKs) signaling pathways, where βarrestins serve as scaffolds for multiprotein complexes assembled on activated phosphorylated GPCRs [40]. Our results indicate that CLZ upregulates the expression of both βarrestin1 mRNA and proteins and that this effect correlates with increased ERK1/2 protein levels. However, we observed a decrease in the Grk2 level (Figure 3). It is known that interaction with Grk2-phosphorylated GPCRs is a requirement for the activity of βarrestins, which regulate downstream signaling pathways, e.g., ERK1/2, and sterically block interactions with heterotrimeric G proteins, leading to rapid desensitization of G protein-mediated signaling cascades [41] (Figure 5). However, it appears that other GRKs, e.g., Grk5 or 6, may also be involved in the phosphorylation of GPCRs [42]. Therefore, the ERK1/2 activation process can take place in a Grk2-independent manner. One possibility is that different Grks phosphorylate distinct sites on the C tail and inner loops of the receptor. This might, in effect, set up a “bar code” that somehow instructs βarrestin (by inducing distinct conformational changes) on its downstream functions [43]. It is worth mentioning that Grk2 is emerging as a signal transducer itself, being able to interact with a variety of non-GPCR proteins [41]. On the other hand, a lack of βarrestin signaling has been shown in the absence of active G proteins [44]. These facts demonstrate the complexity of the signal transduction system.

CLZ acts through a limited number of receptors. Therefore, when considering which GPCRs might be involved in this signaling, 5HT2A seems to be the most appropriate. It has been shown that CLZ is the most efficacious antipsychotic for activating ERK1/2 kinases. Stimulation of ERK1/2 by CLZ could be mediated by the 5HT2A receptor through the novel mechanism “biased agonism”, even though other cellular targets are involved [46]. Although data indicate a role of the 5HT2A receptor in ERK1/2 activation, our results, obtained by an autoradiographic analysis of [3H]ketanserin binding, showed no significant changes in the level of this receptor in the PFC following CLZ administration. On the other hand, we found that CLZ increased the heterodimerization of 5HT1A-5HT2A receptors (using Proximity Ligation Assay) in the PFC [6] and that this effect may be responsible for activation of the ERK1/2 pathway.

Acute treatment with CLZ has been shown to activate ERK1/2 [47]. However, research by Ahmed et al. (2008) showed that chronic administration of CLZ has a transient effect on the ERK1/2 level. The authors measured the ERK1/2 level 2 and 24 h after the last injection. At 2 h later, an increase in ERK1/2 was observed, while 24 h after the last dose of CLZ, the level of ERK1/2 remained unchanged [48]. These studies also showed no effect of HAL on ERK1/2 expression in any brain region. The increase in ERK1/2 observed by us after acute CLZ administration is consistent with the results of Pereira et al. (2009), who showed that a single administration of CLZ caused a decrease in pERK1 activation after 10 min, while 1 h after CLZ administration, increases in pERK1 and pERK2 were observed [49]. The mechanisms underlying the actions of CLZ on the ERK transduction pathway in the PFC are largely unknown, and the results presented above indicate that ERK activation by CLZ is time- and dose-dependent.

### 3.2. Effect of KET on the GPCRs Signaling Pathway

In the present study, we observed a decrease in *DRD2* gene expression but only after acute KET treatment. However, we did not observe statistically significant changes in DRD2 in the PFC after acute KET administration in a previous study involving a radioligand autoradiographic analysis [6]. Even though our study did not find a relationship between mRNA expression and DRD2 receptor levels, there is evidence to suggest that this receptor mediates the rapid effects of KET [50]. Pretreatment with HAL (a non-selective dopamine DRD2/DRD3 antagonist) but not SCH23390 (a dopamine DRD1 receptor antagonist) significantly prevented the effects of KET or MK-801 in the Forced Swim Test (FST). After sub-chronic administration of KET, we did not observe changes in DRD2 mRNA expression, which may confirm that this receptor, which is located within the PFC, is involved rather in the rapid effects of KET. In contrast, the observed decrease in *DRD2* mRNA expression after HAL administration was due to the strong antagonistic properties of HAL to DRD2. It is interesting, however, that we did not observe a decrease in *DRD2* expression in the group that had previously been treated with KET. This suggests an effect of the interaction between these two compounds on the DRD2 (Figure 2B). In our previous study, we showed that repeated administration of KET altered neither the density nor the expression of mRNA encoding for dopamine DRD2 receptors and that CLZ did not also change these parameters, either when administered separately in the striatum or ventral tegmental area. However, when given in combination through repeated administration of CLZ following repeated administration of KET, an increase in the biosynthesis of presynaptic dopamine DRD2 receptors was observed [51]. The most probable explanation for this might be that KET-induced biochemical alterations are necessary for CLZ action.

Certainly, the most interesting result is the demonstration of changes in βarrestin1 and βarrestin2 after KET administration. After acute and sub-chronic administration of KET, we observed increases in the expression and protein level of βarrestin1. However, the mRNA level of *βarrestin1* was upregulated, while the mRNA level of *βarrestin2* decreased after the sub-chronic administration of KET. While βarrestin1 appears to regulate the desensitization of numerous GPCRs, including DRD1, DRD2, and µ-opioid receptors (µORs) [52], βarrestin2 appears to be more effective for mediating agonist-dependent internalization and for promoting faster recycling of multiple GPCRs [52]. In addition, studies on mice lacking βarrestin1/βarrestin2 showed that the administration of apomorphine reduces locomotor activity in both KO strains [53]. The observed differences in βarrestin1 and βarrestin2 mRNA expression levels are very surprising. It has been shown that mice lacking βarrestin1 show no phenotypical changes [54]. This effect is explained by the homology of these two βarrestins, which are 75% identical. In βarrestin1-deficient mice, it appears that the lack of βarrestin1 can be compensated for through the induction of βarrestin2 expression. Perhaps compensation at the level of expression of these two βarrestins would also be observed after KET treatment. Beyond the glutamate system, KET interacts with several additional neurotransmitter systems, including opioid receptors, and it is currently used as an antinociceptive agent for acute and chronic pain [55]. It has been shown that chronic treatment with µOR agonists results in the development of antinociceptive tolerance, causing a significant increase in the expression of βarrestin2 in the cortex and striatum [56]. The downregulation of βarrestin2 observed in our study after sub-chronic administration of KET results from actions of the opioid system. The lack of confirmation of the result at the protein level seems to be due to the specificity of the antibody used. Furthermore, the µOR, whose role seems to be crucial in the phenomenon described above, was not included in assay study screening.

In our study, we also found that KET decreases the Girk3 level. GIRK channels are activated by a large family of GPCRs that are involved in the key neurological processes, such as neuronal plasticity and learning/memory, and are sensitive to different drugs of abuse, making them relevant targets for behavioral studies related to cognition and drug addiction [57,58]. It has been shown that Girk3 knockout mice do not differ from wild-type mice in tests of open field motor activity and motor coordination. In addition, they have similar agonist-induced currents but show less severe sedative withdrawal and reduced self-administration of cocaine [59]. The GIRK knockout mouse phenotype suggests that Girk3 channels may be involved in drug abuse [60]. The observed effect of KET on Girk3 levels may indicate that this channel mediates the addictive effects of KETs.

Other interesting results observed in the present study concern a significant decrease in the level of ERK1/2 after acute administration of KET, while there was no change in the level of Grk2 after acute treatment with KET. Conversely, after sub-chronic administration of KET, we observed a decrease in the Grk2 level but no changes in ERK1/2. Similar to CLZ, we also observed a time-dependent effect of KET on the Grk2 and ERK2 levels. The changes in ERK1/2 levels appeared very fast. Rame et al. showed a change in the phosphorylation levels of Thr202/Tyr204-ERK, although they observed this effect 30 min after KET administration [61]. The complexity of the observed changes demonstrates how difficult it is to identify relevant biochemical markers of action for these drugs.

## 4. Materials and Methods

### 4.1. Animals

Male C57Bl/6J mice weighing approximately 26 g and 11 weeks of age (Charles River, Germany) were housed in a room with a 12 h light–dark cycle (lights on at 07:30) and constant temperature (21 ± 2 °C) and humidity (40–50%) conditions in standard laboratory cages. Five mice were housed in each cage, and they had access to food and water ad libitum. All experimental procedures were approved and performed in accordance with the Bioethical Committee II at the Maj Institute of Pharmacology, Polish Academy of Sciences, Kraków, Smetna 12, Poland (Number of Bioethical approvals: #1196, 25 June 2015).

Ketamine (KET, ketamine hydrochloride) (20 mg, /kg) and/or clozapine (CLZ, 0.3 or 1 mg/kg, free base) and haloperidol (HAL 0.1 mg/kg, free base) were injected 1 h before decapitation of the mice. KET (10% aqueous solution of 115.34 mg/mL, Biowet, Poland) was dissolved in saline (SAL) to give a concentration of 100 mg/kg. CLZ (Tocris, United Kingdom) was dissolved in 1M hydrochloric acid and then diluted in SAL (to obtain ~pH 6.5–7.0, NaOH solution was added). HAL (Polfa, Warszawa S.A, Poland) was diluted in SAL.

Experiments were carried out using two different paradigms: acute and sub-chronic administration. For the acute paradigm, drugs (SAL, KET, and/or CLZ at 0.3 mg/kg; intraperitoneally (i.p.)) were administered twice (the second administration was 24 h after the first one), and mice were decapitated 1 h after the second injection. This experimental paradigm was in accordance with that used in our previous behavioral studies [5]. In the acute paradigm of treatment, we only tested CLZ in a dose of 0.3 mg/kg because coadministration of KET and CLZ 1 mg/kg causes the sedation of mice. For the sub-chronic paradigm, KET (or SAL) was repeatedly administered (i.p.) for seven consecutive days, followed by replacement with CLZ (0.3 or 1 mg/kg; i.p.) or HAL (0.1 mg/kg; i.p.) for the next 7 days. All injections were performed once per day during this period. Mice were sacrificed 24 h after the last drug administration. The brain tissues were frozen at −80 °C and stored at this temperature until RNA and protein extraction.

### 4.2. RNA and Protein Isolation

The RNA purification procedure was carried out according to the procedure attached to the commercially available kit (RNAeasy Mini Kit, Qiagen, Hilden, Germany). During the purification procedure, the protein was precipitated from lysate with cold acetone (−20 °C) for 2 h and dissolved in urea buffer. The RNA quality was checked by microcapillary electrophoresis (Experion RNA StdSens Analysis Kit, Bio-Rad, Hercules, CA, USA), and the total RNA concentration was measured with a NanoDrop ND-1000 Spectrometer (Thermo Fisher Scientific, Waltham, MA, USA). Samples that passed the quality threshold (RIN > 8.0) were used for further experiments.

### 4.3. RT2 qPCR

The amplified cDNA was diluted with nuclease-free water and added to the RT2 qPCR SYBR Green Master Mix (Thermo Fisher Scientific, Waltham, MA, USA). Twenty-five microliters of the experimental cocktail was added to each well of the RT² Profiler™ PCR Array Mouse GPCR Signaling PathwayFinder (Qiagen, Hilden, Germany). Real-time PCR was performed on the CFX System (Bio-Rad, Hercules, CA, USA). The RT-PCR reactions were carried out with the following cycles: enzyme activation at 95 °C/10 min, 40 cycles of denaturation at 95 °C/15 s, and subsequent annealing/elongation at 60 °C/1 min. The screening analysis was performed on n = 3 individuals per group in the acute group, who received KET at a dose of 20 mg/kg i.p. or CLZ at a dose of 0.3 mg/kg i.p. or both drugs together. The genes that were identified as statistically significant in the RT2 qPCR Array were further verified by RT-PCR analysis using TaqMan Primers (Table 2). The RT-PCR analysis was performed for n = 5 individuals per group, and technical duplicates were used.

### 4.4. Data Analysis of RT2 qPCR and RT-PCR

Each RT2 qPCR array contained 5 separate housekeeping genes (*actb*, *b2m*, *gapdh*, *gusb*, and *hsp90ab1*) that were used for normalization of the sample data. Normalization to the housekeeping genes was performed by calculating the ΔCt for each gene of interest in the plate. Any Ct value > 35 was identified as a negative call. The RT2 Profiler PCR Array data were calculated as the fold change based on the widely used and agreed upon ∆∆Ct method that was first described by Livak Schmittgen [62]. Data from the RT-PCR analysis were also based on the ΔΔCt method with normalization of the raw data to one of the housekeeping genes (*βactin*, *gapdh*, or *18s*).

### 4.5. Western Blot

The protein containing fraction from the prefrontal cortex (PFC) was obtained by the purifying process described above. The concentration of proteins was determined using the Bradford Reagent (Sigma-Aldrich, Saint Louis, MO, USA) in accordance with the manufacturer’s protocol. Equal concentrations of PFC proteins were mixed with 4X Bolt^®^ LDS Sample Buffer (Invitrogen, Waltham, MA, USA) and 10X Bolt^®^ Sample Reducing Agent (Invitrogen) and then denatured at 70 °C for 10 min. Samples were separated on Bolt™ 4–12% Bis-Tris Plus Gels (Invitrogen) under reducing conditions in 20X Bolt^®^ MES SDS Running Buffer (Invitrogen), incubated in 20% ethanol for 10 min, and transferred to immunoblot PVDF membranes (iBlot^®^ 2 Transfer Stacks, nitrocellulose, Invitrogen, Waltham, MA, USA) in accordance with the manufacturer’s protocol. Primary and secondary antibodies were suspended in an iBind™ Solution Kit followed by membrane incubation on iBind™ Cards using the iBind™ Western Device (SLF1000, Invitrogen, Waltham, MA, USA) for 2.5 h or overnight. Primary antibodies for βarrestin1 (ab31868, Abcam, Cambridge, UK), βarrestin2 (ab31294, Abcam, Cambridge, UK), ERK1/2 (sc-514302, Santa Cruz, CA, USA), Grk2 (sc-562, Santa Cruz, CA, USA), and GIRK 3 (APC-038, Alomone Labs, Jerusalem, Israel) were used at a concentration of 1:200. The secondary anti-goat (bovine anti-goat antibody IgG-HRP, sc-2350, Santa Cruz; mouse anti-goat IgG-HRP, sc-2354, Santa Cruz, CA, USA) and anti-rabbit (Goat Anti-Rabbit IgG H&L (HRP), ab6721, Abcam, Cambridge, UK) antibodies were used at concentrations of 1:3000 and 1:4000, respectively. As a loading control, β-Actin (Monoclonal Anti-β-Actin antibody produced in mouse, A5441, Sigma-Aldrich, Saint Louis, MO, USA) was applied at a concentration of 1:2000, and its corresponding secondary antibody (Anti-Mouse IgG, A9044, Sigma-Aldrich, Saint Louis, MO, USA) was applied at a concentration of 1:4000. The electrophoretic bands were detected using the Clarity™ Western ECL Substrate (Bio-Rad, Hercules, CA, USA) and FUJIFILM LAS-4000 (Fujifilm Life Science, USA) device. Blot analysis was performed using ImageJ 1.53e software (Wayne Rusband and NIH, USA). Due to limited gel spots, a minimum of two samples from different groups was included in each blot. Separate blots were made for the samples after CLZ and HAL administration. On each gel, irrespective of the neuroleptic tested, there were always at least two samples from the control and the KET-treated group. Each sample was tested using a minimum of two technical duplicates on two different gels.

### 4.6. Statistical Analysis

All data were compared using the two-way ANOVA where the study factors were KET and the study drugs (CLZ and HAL). In addition, a Tukey multiple comparison test was performed for cases where ANOVA led to the conclusion that there was evidence of differences in group means. GraphPad Prism 8.4.3 was used for the statistical analysis.

## 5. Conclusions

This study aimed to search for biochemical markers of CLZ activity in the context of KET-induced cognitive impairment. The screening kit we used to study the expression of GPCR-related signal transduction allowed us to select several important genes affected by CLZ. However, the obtained data do not explain the mechanism of action of CLZ that is responsible for reversing KET-induced cognitive impairment. Nevertheless, we demonstrated several other interesting aspects of CLZ and KET activity.

## Figures and Tables

**Table 1:** List of genes used for the screening analysis (Mouse GPCR Signaling PathwayFinder Array). Statistically significant genes (*p* < 0.05) are presented in bold.

G-Protein Coupled Receptors (GPCRs)	
Metabotropic Glutamate and GABA-Like Receptors	*Casr*, ***mGLuR1***, ***mGluR2***, *mGluR4*, ***mGluR5***, *mGluR7*
Rhodopsin-Like Receptors	*Adora2a*, *Adrb1*, ***Adrb2***, *Agt*, *Agtr1a*, *Agtr1b*, *Agtr2*, *Agtrap*, *Drd1*, ***Drd2***, ***Galr2***, *Lhcgr*, *Lpar1*, *Lpar2*, *Oprd1*, *Oprk1*, *Ptgdr*, *Rho*, *Tshr*
Secretin-Like Receptors	*Adgrb1*, *Adora2a*, *Calcr*, *Calcrl*, *Crhr1*, ***Crhr2***, *Gcgr*, *Sctr*
Other G-Protein Coupled Receptors (GPCRs)	*Edn1*, *S1pr1*, *S1pr2*, *S1pr3*
**G-Protein Coupled Receptor Signaling Pathways**	
Dopamine Receptor Signaling	*Drd1*, ***Drd2***, *Oprd1*
G-Protein and cAMP/ Protein Kinase A Signaling	*Adcy5*, *Adora2a*, *Adrb1*, ***Adrb2***, *Calcr*, *Calcrl*, *Ccl2 (MCP1)*, *Col1a1*, *Crhr1*, ***Crhr2***, *Cyp19a1*, *Drd1*, *Edn1*, ***Galr2***, *Gcgr*, *Gnaq*, *Gnas*, ***Grm2***, *Grm4*, *Il1r1*, *Il1r2*, *Lhcgr*, *Nos2 (iNos)*, *Oprd1*, *Oprk1*, *Ptgs2 (Cox2)*, *Rgs2*, *Tshr*, *Ucp1*, *Vegfa*
G-Protein and IP3/Phospholipase C Signaling	*Agtr1a*, *Agtr1b*, *Calcrl*, *Casr*, *Edn1*, *Gnaq*, ***Grm5***, *Lpar1*, *Lpar2*, *Pik3cg*
Metabotropic Glutamate Receptor Signaling	***Grm1***, ***Grm2***, *Grm4*, ***Grm5***, *Grm7*
Neuropeptide Signaling	*Adgrb1*, *Oprd1*
Adenosine Receptor Signaling	*Adcy5*, *Adora2a*
Calcium Signaling	*Ccl2 (MCP-1)*, *Ccl4 (Mip-1b)*, *Col1a1*, *Egr1*, *Elk4*, *Kcnh8 (Elk1)*, *Ptgs2 (Cox2)*
PKC Signaling	*Agtr2*, *Col1a1*, *Dusp14*, *Egr1*, *Fgf2 (bFGF)*, *Fos*, *Il1r1*, *Il1r2*, *Jun*, *Junb*, *Mmp9*, *Myc*, *Nos2 (iNos)*, *Prkca*, *Ptgs2 (Cox2)*, *Rgs2*, *Serpine1 (Pai-1)*, *Socs1*
Tyrosine Kinase Signaling	***Adrb2***, *Akt1*, *Ccl4 (Mip-1b)*, *Ccn2*, *Pdpk1*, *Prkca*, *Rgs2*, *Socs1*, *Ywhaz*
Protein Serine/Threonine Kinase Signaling	*Akt1*, *Cdkn1b (p27Kip1)*, *Pdpk1*, *Ptgdr*
MAP Kinase Signaling	***Adrb2***, *Agt*, *Fgf2 (bFGF)*, *Grm1*, *Grm4*, *Prkca*, *Tnf*, *Ucp1*
PI3 Kinase Signaling	*Akt1*, *Bcl2*, *Bcl2l1 (Bcl-XL)*, *Ccnd1*, *Cdkn1b (p27Kip1)*, *Cflar (Casper)*, *Pdpk1*, *Pik3cg*, *Ptgs2 (Cox2)*
Nitric Oxide/cGMP Signaling	*Agtr2*, *Nos2 (iNos)*, *Ptgs2 (Cox2)*, *Tnf*
RHO Signaling	*Ccn2*, *Cdkn1b (p27Kip1)*, *Fos*, *Il1b*, *Ptgs2 (Cox2)*, *Rho*, *Serpine1 (Pai-1)*
IκB Kinase/NFκB Cascade	*Agt*, *Cflar (Casper)*, *Icam1*, *Il1b*, *Il2*, *Max*, *Nos2 (iNos)*, *Ptgs2 (Cox2)*, *Tnf*, *Vcam1*
JAK/STAT Signaling	*Ccl2 (MCP-1)*, *Cdkn1a (p21Cip1*, *Waf1)*, *Socs1*
Other G-Protein Coupled Receptor Signaling Genes	***Arrb1***, ***Arrb2***, *Ccne1*, *Ccne2*

**Table 2:** The selection of genes and accession numbers of the probes used in the RT-PCR study.

Assay ID	Gene Symbol	Description
Mm00438308_m1	*Crhr2*	Corticotropin-Releasing Hormone Receptor 2
Mm00438545_m1	*DRD2*	Dopamine Receptor 2
Mm02524224_s1	*Adrb2*	βeta-2 adrenergic receptor
Mm00617540_m1	*Arrb1*	Arrestin βeta 1
Mm00520666_g1	*Arrb2*	Arrestin βeta 2
Mm00726392_s1	*Galr2*	Galanin Receptor 2
Mm00810219_m1	*mGluR1*	Metabotropic glutamate receptor 1
Mm01235831_m1	*mGluR2*	Metabotropic glutamate receptor 2
Mm00690332_m1	*mGluR5*	Metabotropic glutamate receptor 5
Mm03928990_g1	*Rn18s*	18S ribosomal RNA
Mm99999915_g1	*GAPDH*	Glyceraldehyde-3-phosphate dehydrogenase

**Table 3:** Two-way ANOVA analysis of gene expression after acute treatment. Significant statistical results are indicated in bold: * *p* < 0.05; ** *p* < 0.01.

Gene Symbol	Interaction	KET	CLZ
*βarrestin 1*	**F_(1,16)_ = 11.47;** *p* < 0.01 **	F_(1,16)_ = 3.304; *p* = 0.087	F_(1,16)_ = 0.125; *p* = 0.727
*βarrestin 2*	**F_(1,16)_ = 12.07;** *p* < 0.01 **	F_(1,16)_ = 0.449; *p* = 0.512	F_(1,16_) = 0.108; *p* = 0.746
*Dopamine Receptor 2 (DRD2)*	**F_(1,16)_ = 7.742;** ***p* < 0.05**	**F_(1,16)_ = 5.716;** ***p* < 0.05**	F_(1,16)_ = 0.164; *p* = 0.690
*Metabotropic glutamate receptor 1 (mGluR1)*	F_(1,16)_ = 1.141; *p* = 0.301	F_(1,16)_ = 2.835; *p* = 0.111	**F_(1,16)_ = 5.041;** *p* < 0.05 *
*Metabotropic glutamate receptor 2 (mGluR2)*	F_(1,16)_ = 1.759; *p* = 0.203	F_(1,16)_ = 0.013; *p* = 0.908	F_(1,16)_ = 0.013; *p* = 0.908
*Metabotropic glutamate receptor 5 (mGluR5)*	F_(1,16)_ = 0.8424; *p* = 0.372	F_(1,16)_ = 2.615 *p* = 0.125	**F_(1,16)_ = 15.47;** *p* < 0.01 **
*Galanin Receptor 2*	F_(1,16)_ = 6.699; *p* < 0.05	F_(1,16)_ = 2.450 *p* = 0.137	F_(1,16)_ = 0.018; *p* = 0.892
*βeta-2 adrenergic receptor*	F_(1,16)_ = 0.6512; *p* = 0.431	F_(1,16)_ = 0.366; *p* = 0.553	F_(1,16)_ = 0.026; *p* = 0.873
*Crhr2*	F_(1,16)_ = 1.016; *p* = 0.328	F_(1,16)_ = 0.002; *p* = 0.961	F_(1,16)_ = 0.201; *p* = 0.659

**Table 4:** Two-way ANOVA analysis of gene expression after sub-chronic treatment. Significant statistical results are indicated in bold: * *p* < 0.05; ** *p* < 0.01; *** *p* < 0.001.

Gene Symbol	Interaction	KET	CLZ
*βarrestin 1*	F_(2,24)_ = 1.598; *p* = 0.223	F_(1,24)_ = 2.345; *p* = 0.138	F_(2,24)_ = 2.432; *p* = 0.109
*βarrestin 2*	F_(2,24_) = 0.871; *p* = 0.431	**F_(1,24)_ = 14.41;** *p* < 0.001 ***	F_(2,24)_ = 1.842; *p* = 0.180
*Dopamine Receptor 2 (DRD2)*	F_(2,24)_ = 2.730; *p* = 0.085	F_(1,24)_ = 0.258; *p* = 0.616	F_(2,24)_ = 4.135; *p* = 0.023
*Metabotropic glutamate* *receptor 1 (mGluR1)*	F_(2,24)_ = 0.352; *p* = 0.706	F_(1,24)_ = 0.772; *p* = 0.388	F_(2,24)_ = 0.363; *p* = 0.699
*Metabotropic glutamate * *receptor 2 (mGluR2)*	F_(2,24)_ = 0.698; *p* = 0.507	F_(1,24)_ = 0.174; *p* = 0.679	F_(2,24)_ = 2.308; *p* = 0.121
*Metabotropic glutamate * *receptor 5 (mGluR5)*	F_(2,24)_ = 0.232; *p* = 0.794	F_(1,24)_ = 2.770; *p* = 0.109	F_(2,24)_ = 3.045; *p* = 0.066
*Galanin Receptor 2*	F_(2,24)_ = 0.778; *p* = 0.470	F_(1,24)_ = 0.024; *p* = 0.877	F_(2,24)_ = 0.781; *p* = 0.469
*βeta-2 adrenergic receptor*	F_(2,24)_ = 1.018; *p* = 0.376	F_(1,24)_ = 1.229; *p* = 0.278	F_(2,24)_ = 1.748; *p* = 0.195
*Crhr2*	F_(2,24_) = 0.390; *p* = 0.681	F_(1,24)_ = 0.002; *p* = 0.960	F_(2,24)_ = 1.824; *p* = 0.183
**Gene Symbol**	**Interaction**	**KET**	**HAL**
*βarrestin 1*	F_(1,16)_ = 3.912; *p* = 0.065	F_(1,16)_ = 0.423; *p* = 0.524	F_(1,16)_ = 0.097; *p* = 0.758
*βarrestin 2*	**F_(1,16)_ = 10.800;** *p* < 0.01 **	F_(1,16)_ = 3.856; *p* = 0.067	F_(1,16)_ = 2.764; *p* = 0.115
*Dopamine Receptor 2 (DRD2)*	**F_(1,16)_ = 6.586;** *p* < 0.05 *	F_(1,16)_ = 0.846; *p* = 0.371	**F_(1,16)_ = 9.375;** *p* < 0.01 **
*Metabotropic glutamate * *receptor 1 (mGluR1)*	F_(1,16)_ = 0.676; *p* = 0.423	F_(1,16)_ = 1.289; *p* = 0.273	F_(1,16)_ = 0.005; *p* = 0.939
*Metabotropic glutamate * *receptor 2 (mGluR2)*	F_(1,16)_ = 0.169; *p* = 0.674	F_(1,16)_ = 1.238; *p* = 0.282	F_(1,16)_ = 4.453; *p* = 0.051
*Metabotropic glutamate * *receptor 5 (mGluR5)*	F_(1,16)_ = 3.583; *p* = 0.076	F_(1,16)_ = 0.714; *p* = 0.411	F_(1,16)_ = 0.0701; *p* = 0.795
*Galanin Receptor 2*	F_(1,16)_ = 1.508; *p* = 0.237	F_(1,16)_ = 2.089; *p* = 0.168	F_(1,16)_ = 0.397; *p* = 0.537
*βeta-2 adrenergic receptor*	F_(1,16)_ = 0.131; *p* = 0.722	F_(1,16)_ = 4.120; *p* = 0.059	F_(1,16)_ = 4.758; *p* = 0.054
*Crhr2*	F_(1,16)_ = 0.196; *p* = 0.663	F_(1,16)_ = 0.069; *p* = 0.796	F_(1,16)_ = 2.714; *p* = 0.119

**Table 5:** Western blot statistical analysis after acute treatment. Significant statistical results are shown in bold: ** *p* < 0.01, *** *p* < 0.0001.

Protein Symbol	Interaction	KET	CLZ
βarrestin 1	**F_(1,16)_ = 41.21;** *p* < 0.001 ***	F_(1,16)_ = 0.2384; *p* = 0.632	F_(1,16)_ = 0.918; *p* = 0.352
βarrestin 2	F_(1,16)_ = 0.024; *p* = 0.878	F_(1,16)_ = 0.048; *p* = 0.828	**F_(1,16)_ = 17.87;** *p* < 0.001 ***
Grk2	**F_(1,16)_ = 2.523;** ***p* = 0.132**	F_(1,16)_ = 2.409; *p* = 0.140	F_(1,16)_ = 23.35; *p* < 0.01 **
Erk1/2	F_(1,16)_ = 0.261; *p* = 0.616	**F_(1,16)_ = 12.480;** *p* < 0.001 **	F_(1,16)_ = 2.825; *p* = 0.112
Girk 3	F_(1,16)_ = 2.750; *p* = 0.117	**F_(1,16)_ = 36.42;** *p* < 0.0001 ***	F_(1,16)_ = 0.487; *p* = 0.495

**Table 6:** Two-way ANOVA analysis of protein levels in the blot after sub-chronic treatment. * *p* < 0.05, *** *p* < 0.001.

**Protein Symbol**	**Interaction**	**KET**	**CLZ**
βarrestin 1	F_(2,24)_ = 1.200; *p* = 0.318	F_(1,24)_ = 2.709; *p* = 0.112	F_(2,24)_ = 2. 654; *p* = 0.091
βarrestin 2	F_(2,24)_ = 0.172; *p* = 0.843	F_(1,24)_ = 0. 255; *p* = 0.618	F_(2,24)_ = 1.165; *p* = 0.329
Grk2	F_(2,24)_ = 1.471; *p* = 0.249	**F_(1,24)_ = 5.516;** *p* = 0.027 *	F_(2,24)_ = 2.062; *p* = 0.149
Erk1/2	F_(2,24)_ = 1.061; *p* = 0.361	F_(1,24)_ = 0.126; *p* = 0.725	F_(2,24)_ = 0.721; *p* = 0.200
Girk 3	F_(2,24)_ = 1.900; *p* = 0.171	F_(1,24)_ = 0.586; *p* = 0.431	F_(2,24)_ = 0.871; *p* = 0.431
**Protein Symbol**	**Interaction**	**KET**	**HAL**
βarrestin 1	**F_(1,16)_ = 19.77;** *p* = 0.0004 ***	F_(1,16)_ = 1.183; *p* = 0.2928	F_(1,16)_ = 0.404; *p* = 0.533
βarrestin 2	F_(1,16)_ = 0.324; *p* = 0.577	F_(1,16)_ = 0.045; *p* = 0.833	F_(1,16)_ = 1.876; *p* = 0.189
Grk2	**F_(1,16)_ = 6.290;** *p* = 0.023 *	F_(1,16)_ = 0.784; *p* = 0.389	**F_(1,16)_ = 5.180;** *p* = 0.037 *
Erk1/2	F_(1,16_) = 0.437; *p* = 0.518	F_(1,16)_ = 0.624; *p* = 0.441	F_(1,16)_ = 3.602; *p* = 0.076
Girk 3	F_(1,16)_ = 0.853; *p* = 0.369	F_(1,16)_ = 1.266; *p* = 0.277	F_(1,16)_ = 0.137; *p* = 0.740

## Data Availability

Data supporting the reported results are available on request from the corresponding author.
